# 

**Published:** 1868-06-15

**Authors:** 


*CONTENTS:*

ORIGINAL PAPERS:

Progressive Paralysis of the Insane. By A. W. Bosworth, M. A., Paris, (concluded.) .... 379
Fluid Extracts .... 393
Cincinnati Correspondence .... 396
A Case of Traumatic Tetanus successfully treated with the Calabar Bean. By A. J. Baxter, M. D., Chicago, Ill. .... 398
Treatment of Diphtheria. By James C. Bascomb, M. D., McLean, Ill. .... 405
A Case of Mistaken Diagnosis. By Wm. L. Coe, M. D., Morrison, Ill. .... 406

BOOK NOTICES:

Materia Medica for the use of Students .... 408
Contributions relating to the Causation and Prevention of Disease, and to Camp Diseases .... 408
The American Journal of Obstetrics and Diseases of Women and Children .... 409

EDITORIAL .... 409
PROCEEDINGS OF SOCIETIES .... 410

